# Supplementary material for: Burnout in French General Practitioners: A Nationwide Prospective Study
Source: Int J Environ Res Public Health. 2021 Nov 16;18(22):12044. doi: 10.3390/ijerph182212044 (PMC8624683; doi:10.3390/ijerph182212044)
Supplement: Supplementary file 1 [file ijerph-18-12044-s001.zip › ijerph-1433742-supplementary.pdf]

Table S1: Burnout, by sociodemographic and occupational characteristics (by 100% total in line)

		Level of burnout				
	Total sample	No burnout	Low burnout	Intermediate burnout	Severe burnout	p-value
	n (%) or mean±SD	n (%) or mean±SD	n (%) or mean±SD	n (%) or mean±SD	n (%) or mean±SD	
**Total**	1926 (100%)	1063 (55.2%)	502 (26.1%)	268 (13.9%)	93 (4.8%)	**<0.001**
**Sociodemographic**						
**Gender**						
Female	916 (100%)	540 (58.9%)	234 (25.6%)	111 (12.1%)	31 (3.4%)	**<0.001**
Male	1004 (100%)	521 (51.9%)	267 (26.6%)	154 (15.3%)	62 (6.2%)	
**Age**						
mean	50.0 ± 10.7	49.7 ± 11.2	50.2 ± 10.5	49.8 ± 9.8	52.5 ± 8.6	0.098
<50 years old	840 (100%)	488 (58.1%)	204 (24.3%)	118 (14%)	30 (3.6%)	**0.033**
≥50 years old	1080 (100%)	573 (53.1%)	297 (27.5%)	148 (13.7%)	62 (5.7%)	
**Marital status**						
Single	89 (100%)	37 (41.6%)	29 (32.6%)	18 (20.2%)	5 (5.6%)	0.214
Married or in couple	1644 (100%)	920 (55.9%)	425 (25.9%)	223 (13.6%)	76 (4.6%)	
Divorced, Separated or Widowed	187 (100%)	105 (56.1%)	45 (24.1%)	26 (13.9%)	11 (5.9%)	
**Parenthood**						
Yes	1709 (100%)	958 (56.1%)	433 (25.3%)	235 (13.7%)	83 (4.9%)	0.125
No	211 (100%)	101 (47.9%)	67 (31.8%)	33 (15.6%)	10 (4.7%)	
**Career characteristics**						
**Career length**						
mean	20.7 ± 11.2	20.5 ± 11.6	20.9 ± 11.2	20.4 ± 10	23 ± 9.4	0.222
<20 years	807 (100%)	467 (%)	200 (%)	112 (%)	28 (%)	0.059
≥20 years	1087 (100%)	584 (%)	292 (%)	148 (%)	63 (%)	

**Further qualifications**						
Yes	1219 (100%)	665 (56.2%)	329 (25.5%)	174 (13.4%)	51 (4.9%)	0.624
No	952 (100%)	537 (54.2%)	245 (26.3%)	124 (14.7%)	46 (4.8%)	
**Setting characteristics**						
**Setting location**						
Rural	427 (100%)	238 (55.5%)	113 (26.6%)	67 (15.8%)	9 (2.1%)	**0.008**
Suburban	746 (100%)	398 (53.4%)	192 (25.6%)	103 (13.8%)	53 (7.2%)	
Urban	743 (100%)	422 (56.9%)	193 (25.9%)	97 (13.1%)	31 (4.1%)	
**Commuting time (min)**						
mean	11.8 ± 11.3	11.3 ± 10.5	12.3 ± 11.1	12.8 ± 14.1	12.9 ± 12.4	0.088
<12 minutes	1184 (100%)	668 (56.4%)	303 (25.6%)	156 (13.2%)	57 (4.8%)	0.527
≥12 minutes	722 (100%)	384 (53.2%)	195 (27%)	108 (14.9%)	35 (4.9%)	
**Social practice context**						
**Group practice**						
Yes	657 (100%)	725 (58.4%)	321 (25.8%)	151 (12.2%)	45 (3.6%)	**<0.001**
No	1242 (100%)	325 (49.4%)	172 (26.2%)	116 (17.7%)	44 (6.7%)	
**Home visits**						
Yes	160 (100%)	970 (55.8%)	451 (25.9%)	248 (14.2%)	71 (4.1%)	**<0.001**
No	1740 (100%)	80 (50%)	43 (26.8%)	19 (11.9%)	18 (11.3%)	
**Resident training**						
Yes	608 (100%)	384 (63.2%)	136 (22.4%)	71 (11.7%)	17 (2.7%)	**<0.001**
No	1300 (100%)	671 (51.6%)	359 (27.6%)	194 (14.9%)	76 (5.9%)	
**Work support**						
**Administrative support**						
Yes. on site or by phone	1436 (100%)	813 (%)	376 (%)	183 (%)	64 (%)	**0.050**
No	441 (100%)	229 (%)	111 (%)	78 (%)	23 (%)	
**Partly salaried**						
Yes	283 (100%)	162 (57.2%)	73 (25.8%)	34 (12.1%)	14 (4.9%)	0.755

No	1632 (100%)	895 (55%)	424 (25.9%)	234 (14.3%)	79 (4.8%)	
**Workload**						
**Weekly hours**						
Mean	50.6 ± 12.4	49.1 ± 12	51 ± 11.9	54.4 ± 13.4	53.7 ± 13	**<0.001**
<50 hours/weeks	805 (100%)	503 (62.5%)	196 (24.3%)	77 (9.6%)	29 (3.6%)	**<0.001**
≥50 hours/weeks	1115 (100%)	558 (50%)	305 (27.3%)	189 (17%)	63 (5.7%)	
**Number of daily consultations**						
Mean	28.6 ± 11.1	27.7 ± 9.8	28.9 ± 10.3	30.6 ± 15.9	32 ± 10.5	**<0.001**
<28 consultations/day	961 (100%)	569 (%)	243 (%)	117 (%)	32 (%)	**<0.001**
≥28 consultations/day	915 (100%)	471 (%)	244 (%)	143 (%)	57 (%)	
**Emergency activity**						
**Emergency work**						
Yes	1166 (100%)	639 (54.8%)	309 (26.5%)	166 (14.2%)	52 (4.5%)	0.673
No	750 (100%)	419 (55.9%)	188 (25.2%)	102 (13.6%)	41 (5.5%)	
**Emergency work regulated by EMD**						
Yes	1033 (100%)	563 (54.5%)	278 (26.9%)	148 (14.3%)	44 (4.3%)	0.650
No	129 (100%)	76 (58.9%)	30 (23.3%)	16 (12.4%)	7 (5.4%)	
**Night shifts**						
Yes	221 (100%)	121 (54.7%)	55 (24.9%)	32 (14.5%)	13 (5.9%)	0.638
No	944 (100%)	518 (54.9%)	254 (26.9%)	134 (14.2%)	38 (4%)	
